# The Human Organism as an Integrated Interaction Network: Recent Conceptual and Methodological Challenges

**DOI:** 10.3389/fphys.2020.598694

**Published:** 2020-12-21

**Authors:** Klaus Lehnertz, Timo Bröhl, Thorsten Rings

**Affiliations:** ^1^Department of Epileptology, University of Bonn Medical Centre, Bonn, Germany; ^2^Helmholtz Institute for Radiation and Nuclear Physics, University of Bonn, Bonn, Germany; ^3^Interdisciplinary Center for Complex Systems, University of Bonn, Bonn, Germany

**Keywords:** complex networks, time-series-analysis techniques, surrogate concepts, inverse problem, physiological systems, organ communications, network physiology

## Abstract

The field of Network Physiology aims to advance our understanding of how physiological systems and sub-systems interact to generate a variety of behaviors and distinct physiological states, to optimize the organism's functioning, and to maintain health. Within this framework, which considers the human organism as an integrated network, vertices are associated with organs while edges represent time-varying interactions between vertices. Likewise, vertices may represent networks on smaller spatial scales leading to a complex mixture of interacting homogeneous and inhomogeneous networks of networks. Lacking adequate analytic tools and a theoretical framework to probe interactions within and among diverse physiological systems, current approaches focus on inferring properties of time-varying interactions—namely strength, direction, and functional form—from time-locked recordings of physiological observables. To this end, a variety of bivariate or, in general, multivariate time-series-analysis techniques, which are derived from diverse mathematical and physical concepts, are employed and the resulting time-dependent networks can then be further characterized with methods from network theory. Despite the many promising new developments, there are still problems that evade from a satisfactory solution. Here we address several important challenges that could aid in finding new perspectives and inspire the development of theoretic and analytical concepts to deal with these challenges and in studying the complex interactions between physiological systems.

## 1. Introduction

**Figure 1:**
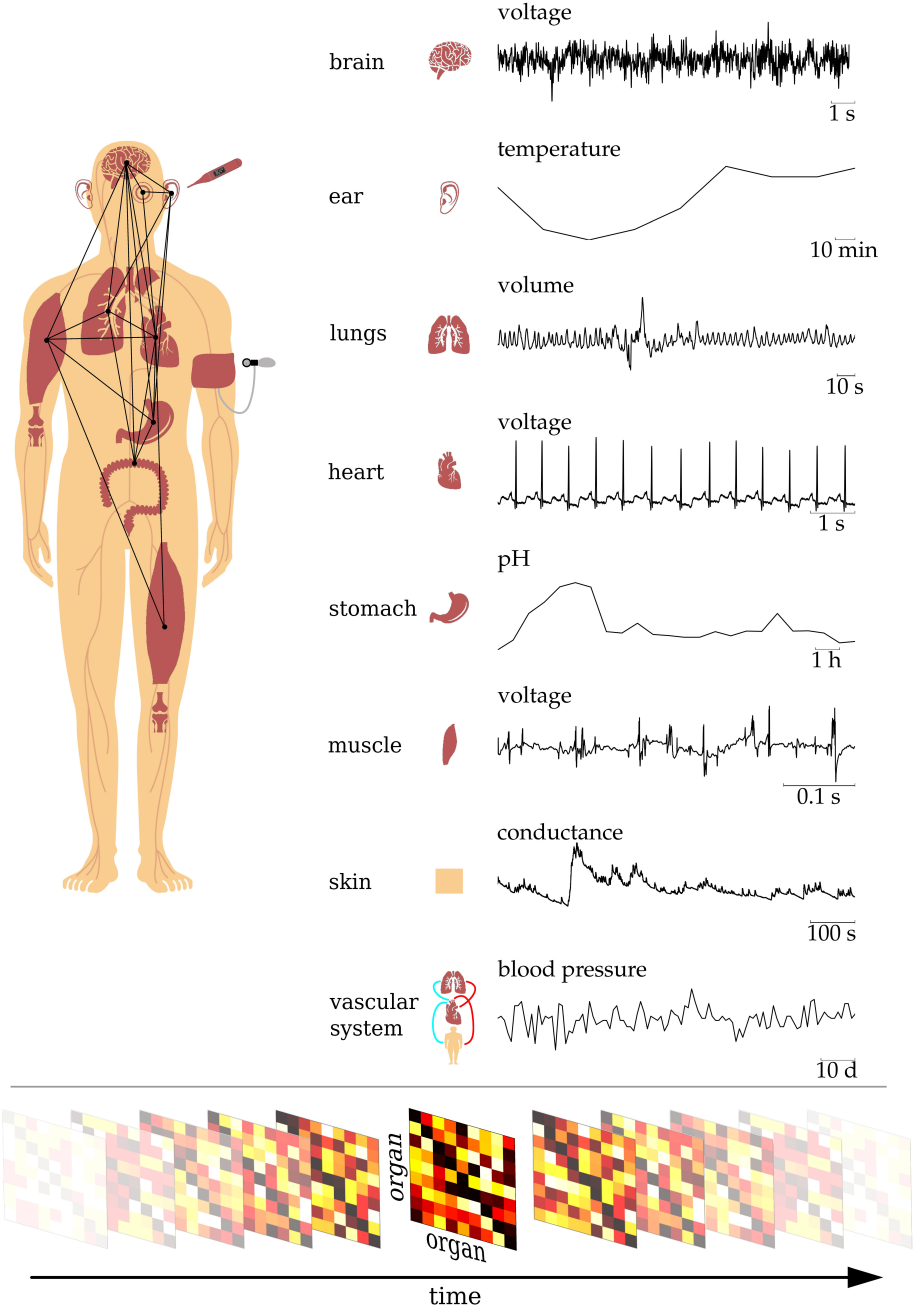
Schematic of the human organism as an evolving complex network of dynamical interactions between organ systems. The dynamics of different organs exhibit a broad range of timescales, and physiological observables are typically based on different physical and/or chemical quantities. Time-dependent organ-organ interaction matrices are derived from a time-resolved time-series-analysis-based characterization of interactions from all pairs of observables. These matrices represent a network that evolves in time, with nodes representing organs and edges representing time-varying interactions between them.

Network physiology (Bartsch et al., 2012, 2015; Bashan et al., 2012; Ivanov et al., 2016) is a novel transdisciplinary research approach that focuses on how physiological systems and subsystems interact, thereby complementing the traditional approaches from systems biology and integrative physiology. Conceptually, it considers the human organism as an evolving complex network—a radically reduced description where the full system is described by an *interaction network*, whose vertices represent distinct physiological subsystems and whose edges represent time-dependent, observation-derived interactions between them (see Figure 1). This reduced description has been utilized in a number of scientific disciplines, and research over the last two decades has demonstrated that the network paradigm can advance our understanding of natural and man-made complex dynamical systems (see e.g., Boccaletti et al., 2006, 2014; Arenas et al., 2008; Barthélemy, 2011; Holme and Saramäki, 2012; Bassett and Sporns, 2017; Halu et al., 2019 for an overview). Although encouraging, the data-driven network approach to the human organism faces a number of challenges. Conceptually, the inference of interactions from observation of the organism's dynamics constitutes a fundamental inverse problem, which has no unique solution (von Helmholtz, 1853). State-of-the-art reconstruction methods require access to a model of the human organism or dynamical data at a preciseness that is not available. Another and more practicable path that is often taken in the network sciences, including network physiology, encompasses (i) a time-series-analysis-based characterization of interactions between all pairs of subsystems, (ii) a derivation of a network from estimated characteristics, and (iii) a characterization of the network with methods from graph theory. In the following, we discuss important challenges of this path from pairwise interactions to interaction networks.

## 2. Challenges With Characterizing Interactions

The characterization of interactions between physiological systems faces several challenges:

- We often do not know exactly the systems' equations of motion;
- We lack knowledge as to how to merge/combine these equations (e.g., due to the issue of time-scale matching);
- We may have insufficient knowledge about relevant structural connections;
- We may not have direct access to interactions between systems (e.g., via probing).

Due to these (and possibly other) limitations, usually linear and non-linear time-series-analysis techniques are employed to quantify interaction properties from pairs of time series of appropriate system observables. Since interactions can manifest themselves in various aspects of the dynamics, analysis techniques originate from diverse fields such as statistics, synchronization theory, non-linear dynamics, information theory, statistical physics, and from the theory of stochastic processes (for an overview, see Pikovsky et al., 2001; Kantz and Schreiber, 2003; Reinsel, 2003; Pereda et al., 2005; Hlaváčková-Schindler et al., 2007; Marwan et al., 2007; Friedrich et al., 2011; Lehnertz, 2011; Lehnertz et al., 2014; Müller et al., 2016; Stankovski et al., 2017; Tabar, 2019). Interactions may impact on amplitudes, phases, frequencies, or even combinations thereof and for some cases it might be more efficient to consider interactions as flow of information. Beyond that, a more detailed characterization of interactions can in general be achieved with state-space-based approaches and with approaches that even allow for interactions in the stochastic (rather than the deterministic) part of the dynamics (Prusseit and Lehnertz, 2008; Rydin Gorjão et al., 2019).

### 2.1. Data-Driven Assessment of Pairwise Interaction Properties

Common linear time-series-analysis techniques (Carter, 1987) such as estimating the linear correlation coefficient, cross-correlation and cross-spectral functions as well as (linear) partial coherence are often used but can mostly provide information about the strength of an interaction since correlation does not imply causation. Linear indices for the direction of an interaction are usually based on the concepts of Granger causality (Seth et al., 2015) or partial directed coherence (Baccalá and Sameshima, 2001; Schelter et al., 2006b) that make use of parametric approaches to estimate (single and joint) properties of the power spectra (Lütkepohl, 2005). Note that linear approaches to characterize interactions are mostly based on amplitudes, and these approaches may not adequately account for the well-known non-linearities in physiological systems (Elbert et al., 1994; West, 2012).

Common non-linear time-series-analysis techniques can be subdivided into two main categories depending on the underlying concept for interaction: synchronization-based (SB) techniques (Pikovsky et al., 2001; Boccaletti et al., 2002; Stankovski et al., 2017) and information-theory-based (IB) techniques (Hlaváčková-Schindler et al., 2007; Amblard and Michel, 2013). SB techniques aim at assessing aspects of generalized synchronization (Čenys et al., 1991; Rulkov et al., 1995; Arnhold et al., 1999) or of phase synchronization (Huygens, 1673; Rosenblum et al., 1996). For generalized synchronization, at first the state spaces of the systems need to be reconstructed from time series of system observables (Kantz and Schreiber, 2003). This allows one to exploit various geometric or dynamic properties to quantify strength and direction of interactions (Arnhold et al., 1999; Pikovsky et al., 2001; Boccaletti et al., 2002; Marwan et al., 2007; Faes et al., 2008; Chicharro and Andrzejak, 2009). For phase synchronization, phase time series of the systems need to be derived from the time series of observables, and there are various approaches that allow one to extract phases from noisy broadband signals (see e.g., Bruns, 2004; Kralemann et al., 2008; Schwabedal and Kantz, 2016). The strength of interactions can then be estimated by exploiting phase differences (Tass et al., 1998; Lachaux et al., 1999; Mormann et al., 2000), and the direction of interactions can be quantified via a phase modeling approach (Rosenblum and Pikovsky, 2001; Smirnov, 2014). Recently, methods have been developed that allow the detection and reconstruction of coupling functions from measured data (Stankovski et al., 2017; Pietras and Daffertshofer, 2019; Rosenblum and Pikovsky, 2019; Bick et al., 2020). IB techniques aim at identifying common information contained in the systems' time series of observables as this would allow one to infer the direction of interaction (“causal relationships”) between systems (Schreiber, 2000; Staniek and Lehnertz, 2008; Vicente et al., 2011; Smirnov, 2014; Timme et al., 2014; Porta and Faes, 2015; Runge, 2018). Note that these techniques are only occasionally used to infer the strength of interaction (e.g., Liu, 2004; Monetti et al., 2009; Jafri et al., 2016), and we are missing techniques to detect and reconstruct coupling functions.

Despite the different concepts and the many time-series-analysis techniques, a discussion about their relative merit lasting for more than 15 years indicates that there is probably no single approach which is best suited to characterize properties of interactions between physiological systems (Smirnov and Andrzejak, 2005; Ansari-Asl et al., 2006; Kreuz et al., 2007; Paluš and Vejmelka, 2007; Smirnov et al., 2007; Osterhage et al., 2007a,b, 2008; Vejmelka and Paluš, 2008; Wendling et al., 2009; Florin et al., 2011; Wang et al., 2014; Zhou et al., 2014; Hirata et al., 2016; Stokes and Purdon, 2017; Xiong et al., 2017; Barnett et al., 2018; Beauchene et al., 2018; Dhamala et al., 2018; Krakovská et al., 2018; Bakhshayesh et al., 2019).

### 2.2. Current Limitations to a Data-Driven Assessment of Pairwise Interaction Properties

Conceptually, the majority of the aforementioned time-series-analysis techniques assumes the investigated systems to be stationary (or at least approximately stationary) and the interactions to be stable and persisting throughout the observation time. By their very nature, however, physiological systems are inherently non-stationary (Marmarelis, 2012) and interactions between them are mostly transient. In some cases, even multiple forms of couplings can coexist (Bartsch et al., 2014; Klimesch, 2018). So far, only a few time-series-analysis techniques were developed to characterize transient interactions between pairs of systems (Hesse et al., 2003; Andrzejak et al., 2006; Faes et al., 2008; Wagner et al., 2010; Hempel et al., 2011; Lehnertz, 2011; Martini et al., 2011; Bartsch et al., 2012; Ma et al., 2014; Liu et al., 2015; Lin et al., 2016; Kostoglou et al., 2019), and it is not yet clear whether there is one single approach that is best suited to characterize all relevant properties of transient interactions between non-stationary physiological systems.

Most physiological systems operate on very different time scales (an der Heiden, 1979; Batzel and Kappel, 2011; Gosak et al., 2018) (cf. Figure 1), and due to distance- and function-related characteristic features, delayed interactions need to be taken into account. The exact delay between physiological systems is usually not known a priori and may be time-dependent. Time-series-analysis techniques designed to characterize delayed interactions thus make use of exhaustive/brute force search methods to identify potential delay(s) (Müller et al., 2003; Silchenko et al., 2010; Dickten and Lehnertz, 2014; Faes et al., 2014; Ye et al., 2015; Lin et al., 2016; Coufal et al., 2017; Ma et al., 2017; Li et al., 2018; Rosinberg et al., 2018). The, in general, high computational burden may limit real-time analyses of delayed interactions. Addressing the issue of different time scales, methods have been proposed recently that aim at a multiscale description of interacting systems (Lungarella et al., 2007; Ahmed and Mandic, 2011; Humeau-Heurtier, 2016; Faes et al., 2017; Paluš, 2019; Jamin and Humeau-Heurtier, 2020).

Interpreting findings from pairwise interaction measurements is a challenging task. Among others, statistical fluctuations and systematic errors may impinge on findings of some interaction property. Moreover, misapplying or misinterpreting time-series-analysis techniques may lead to inappropriate conclusions. Surrogate testing is a crucial tool to ensure the reliability of the results (Schreiber and Schmitz, 2000). Nevertheless, although extensions and new development of surrogate techniques can help to avoid misinterpretations about the strength of an interaction (Andrzejak et al., 2003; Lancaster et al., 2018; Ricci et al., 2019), causal relationships are notoriously difficult to identify (Mayr, 1961; Laland et al., 2011). Although some approaches have been proposed to test the significance of directionality indices (Thiel et al., 2006; Romano et al., 2009; Faes et al., 2010; Jelfs and Chan, 2017), we still lack reliable surrogate techniques for directionality indices as well as for techniques to detect and characterize coupling functions.

## 3. Challenges With Deriving and Characterizing an Integrated Network of Physiological Subsystems

Network physiology considers the human organism as an integrated network, whose vertices are associated with distinct physiological subsystems (i.e., different organs) and edges represent time-varying interactions between vertices. This initial assignment of vertices and edges can have major implications on how an integrated network of interacting physiological subsystems is configured and interpreted (Butts, 2009; Bialonski et al., 2010; Hlinka et al., 2012; Timme and Casadiego, 2014; Wens, 2015; Papo et al., 2016; Nitzan et al., 2017), and a number of challenges arise when identifying and quantifying networks of diverse subsystems with different types of interactions.

### 3.1. Vertices

The definition of vertices of the spatially extended dynamical system *human organism* is notoriously difficult. Although the assignment of vertices to distinct physiological subsystems appears rather intuitive, in practice, vertices are usually associated with sensors that are assumed to be placed such that they sufficiently capture the dynamics of subsystems. This ansatz, which is often not even questioned, requires appropriate spatial and temporal sampling strategies, insights into the physical processes and the statistical properties of the system. Identifying adequate sampling strategies is closely related to issues such as accessibility and non-invasiveness and, more importantly, to what is actually a good observable for a given organ to allow insights into the relevant physical processes. Often used physiological observables range from electric and/or magnetic fields to thermodynamic properties such as temperature, pressure, or volumes as well as to chemical properties such as pH or concentration (cf. Figure 1). Observables often dictate the type of sensor, and there might be limitations concerning their size, positioning, or combinability. Due to their very nature, physiological observables can capture vastly different timescales, ranging from milliseconds to days and months, and we lack appropriate concepts and analysis techniques to match these timescales. Recordings of observables are typically noisy and prone to technical and physiological artifacts.

For single organs, there exists a large number of guidelines and recommendations for the sampling of their activities (e.g., Camm et al., 1996; Kligfield et al., 2007; Seeck et al., 2017; Harford et al., 2019; Tankisi et al., 2020). Nevertheless, with the development of novel sensing technologies (Andreu-Perez et al., 2015), guidelines and recommendations are often challenged (Trägårdh et al., 2006; Garćıa-Niebla et al., 2009; Xia et al., 2012; Grover and Venkatesh, 2016), and by now, we lack commonly accepted guidelines for the spatial and temporal sampling of interactions between different organs to allow insights into the relevant physical processes and the statistical properties of the human organism.

An alternative ansatz, which is often pursued in the neurosciences and in cardiology, would consist in replacing estimations of interaction properties in *sensor-space* with those in *source-space* (see e.g., Van Mierlo et al., 2019 and references therein). This approach requires localizing the sources of electric/magnetic activities that generate the potentials/fields that can be recorded non-invasively on the surface of the body. It constitutes another inverse problem with early explorations dating back to the 1950s using electric field theory. The lack of a unique solution to this inference problem is reflected by a large set of analysis methods that were developed since then to find an appropriate approximation (Jatoi et al., 2014).

### 3.2. Edges

A natural way to define edges of the networked human organism would be to relate them to structural connections within and between physiological subsystems (e.g., synapses, nerve tracts, or the lymph or blood stream). Since we lack non-invasive access to these *structural* edges and their dynamics, a widely used ansatz is to infer *functional* edges via a data-driven assessment of pairwise interaction properties from the subsystems' dynamics using the aforementioned time-series-analysis techniques in an attempt to elucidate the underlying coupling mechanisms (cf. Figure 1). Note that there are by now no commonly accepted genuine multivariate approaches to assess interactions properties from the dynamics of more than two physiological subsystems. Moreover, the assessment may be hampered by the as yet unsolved problem to reliably distinguish between direct and indirect interactions, with the latter being mediated by another—even unobserved—(sub-)system. This can lead to serious misinterpretations of possible causal relationships. The severity of this issue is expressed in a large number of time-series-analysis techniques—based on partialization analysis—that have been proposed over the last two decades to overcome this problem of transitivity (see e.g., Langford et al., 2001; Eichler et al., 2003; Chen et al., 2004; Schelter et al., 2006a,b; Frenzel and Pompe, 2007; Smirnov and Bezruchko, 2009; Vakorin et al., 2009; Nawrath et al., 2010; Jalili and Knyazeva, 2011; Zou et al., 2011; Runge et al., 2012; Stramaglia et al., 2012; Kugiumtzis, 2013; Leistritz et al., 2013; Ramb et al., 2013; Kralemann et al., 2014; Elsegai et al., 2015; Faes et al., 2015; Mader et al., 2015; Zhao et al., 2016; Leng et al., 2020; Marinazzo et al., 2012). All these techniques involve estimating interaction properties between two systems, holding constant the external influences of a third. Their efficiency, however, is severely limited by volume conduction effects, asymmetric signal-to-noise ratios (Albo et al., 2004; Nolte et al., 2004; Xu et al., 2006) as well as by the number of interacting subsystems and the density of connections between them (Rubido et al., 2014; Zerenner et al., 2014; Rings and Lehnertz, 2016).

Spurious indications of strength and direction of interactions can be considered as another related issue which can lead to severe misinterpretations. These indications can result from an instantaneous mixture of activities, i.e., a common source, which may be caused by, e.g., a too close spatial sampling of some organ with multiple sensors. Likewise, it may be due to an—often unavoidable—referential recording as in case of measurements of an organ's electric fields. While a number of proposed extensions to and modification of particularly phase-based time-series-analysis techniques (Stam et al., 2007; Vinck et al., 2011; Stam and van Straaten, 2012; Hardmeier et al., 2014) appear to be less affected by such influences, their general suitability, however, continues to be matter of debate (Yu and Boccaletti, 2009; Peraza et al., 2012; Gordon et al., 2013; Porz et al., 2014; Colclough et al., 2016).

### 3.3. Choosing the Type of Network

Once edges and vertices are defined sufficiently, they are then used to set up a binary or weighted and undirected or directed network, depending on which interaction properties between physiological subsystems have been characterized. An *undirected binary network* characterizes interacting physiological subsystems in terms of connected or disconnected. For such a network, a pair of subsystems is said to be connected by an edge, if an estimated strength of interaction exceeds some threshold. Despite the simplicity of this ansatz, we still lack commonly accepted criteria for the choice of the threshold (Ioannides, 2007; Kramer et al., 2009; Rubinov and Sporns, 2010; Zanin et al., 2012; Fornito et al., 2013).

An *undirected weighted network* characterizes interacting physiological subsystems in terms of how strongly they interact with each other. In such a network, all edges are usually considered to exist, again due to the lack of a reliable definition of a threshold to exclude edges with non-significant interaction strengths. Commonly, the weight of an edge and the estimated strength of an interaction between vertices connected by that edge are set to be equal. While many estimators for the strength of an interaction are normalized, in general, the weight matrix associated with the weighted network is not; hence, it is advisable to suitably normalize this matrix. Furthermore, the distribution of estimated strengths of interaction can have a dominant effect on network properties of interest and need to be taken into account (Ansmann and Lehnertz, 2012; Stahn and Lehnertz, 2017).

Adding information about the direction of interaction to a binary network expands this to a *directed binary network*. As in the undirected case, an appropriately chosen threshold may help to separate significant from non-significant indications of directionality. Even more problematic, the modulus of an estimator for the direction of an interaction typically lacks physical interpretability; often only the sign indicates the direction.

Deriving a *weighted and directed network* by merging both interaction properties—strength and direction—would be preferable, as such a network conveys most information about interacting physiological subsystems. As yet, this task is not solved in a conclusive manner and one needs to keep in mind that strength and direction are distinct but related properties of interactions (Elsegai et al., 2015; Lehnertz and Dickten, 2015; Dickten et al., 2016). While in some specific situations the modulus of an estimator for the direction of an interaction might be interpreted as strength of an interaction, this is not generally valid and has been shown to lead to severe misinterpretations, particularly for uncoupled and for strongly coupled systems (Osterhage et al., 2008; Lehnertz and Dickten, 2015). Both interaction properties should thus be estimated separately but using analysis techniques that based on the same concept (e.g., synchronization theory or information theory). A mixing of different concepts might be ill-advised, as it remains unclear how different concepts translate to each other (Dickten et al., 2016). Moreover, there is no commonly accepted method for how weights should be allocated to an edge's forward and backward direction. While the strength of an interaction has no directionality and is consequently invariant under exchange of vertices, the direction of an interaction is not.

### 3.4. Network Characterization

Graph theory provides a large spectrum of approaches that can be used to characterize an integrated network of physiological subsystems (see e.g., Boccaletti et al., 2006; Arenas et al., 2008; Fortunato, 2010; Barthélemy, 2011; Newman, 2012, for an overview). Characteristics range from *local* ones, which describe properties of network constituents, e.g., individual vertices or edges to *global* ones, which assess properties of the network as a whole. Most characteristics, however, were initially developed for binary networks, and an extension to weighted and/or directed networks is usually not straightforward. As an example, consider the shortest path between two vertices *l* and *k* in a binary network which is the smallest number of edges one has to traverse to reach vertex *l* from vertex *k*. The length of a single path between two vertices in a weighted network is oftentimes defined as the inverse of the edge weight. This definition relies on the observation that the ratio between the weights of two edges equals the ratio between their lengths; other definitions, however, might be equally valid. Influencing factors such as common sources and indirect interactions were shown to impact on the definition of shortest paths (Ioannides, 2007; Bialonski et al., 2011). Similar arguments hold for the clustering coefficient; despite several suggestions for an extension to weighted (Saramäki et al., 2007) and directed networks (Fagiolo, 2007), their suitability for the analysis of an integrated network of physiological subsystems remains to be shown.

Clustering coefficient and mean shortest path are oftentimes used to decide upon a network's small-worldness (Bassett and Bullmore, 2006), and this property has repeatedly been reported for networks from diverse scientific disciplines. Given the many factors that impact on clustering coefficient and mean shortest path, however, these findings continue to be matter of considerable debate (Bialonski et al., 2010; Gastner and Ódor, 2016; Hilgetag and Goulas, 2016; Papo et al., 2016; Hlinka et al., 2017; Zanin et al., 2018).

Since characteristics of networks (as well as of time series from which networks were derived) can be affected by a number of influencing factors, surrogate testing can be applied to eliminate or at least minimize those influences (Schreiber and Schmitz, 2000; Stahn and Lehnertz, 2017). Although such an approach is strongly recommended to avoid severe misinterpretations, we lack surrogate schemes that are appropriate for networks of interacting physiological subsystems and that address the challenges referred to here.

Eventually, an integrated network of physiological subsystems can be regarded as an *evolving network*, whose vertices (and/or edges) change with time. Although it is of utmost importance to understand how the network changes from time step to time step, its investigation requires appropriate methods that would allow a comparison of networks (Mheich et al., 2020). Developing such methods, however, is highly non-trivial, since a network's topological properties necessarily depend on the number of edges and the number of vertices. When both quantities change with time, an unbiased comparison between networks remains difficult.

## 4. Conclusion and Summary

The challenges arising on the path from pairwise interactions to interaction networks call for concerted efforts of all involved communities to advance network physiology. There is an urgent need for sensing concepts and technologies that allow time-locked recordings of relevant physiological observables thereby taking into account their various physical origins as well as their vastly different time scales. Similarly, appropriate concepts and analysis techniques need to be developed to match these time scales and to allow multimodal data fusion (Lahat et al., 2015). Time-series-analysis techniques require further improvements to allow an unambiguous characterization of properties of interactions between more than two systems and under the constraints related to investigating the human organism during (patho-)physiological conditions. Ultimately, the strong heterogeneity of organs and their dynamics calls for better suited network concepts (e.g., based on multilayer/multiplex networks, Boccaletti et al., 2014; Kivelä et al., 2014; Castellani et al., 2016) and possibly requires novel network characteristics and statistical tools. To be successful, these efforts should be scrutinized with the question whether the network framework tells us anything new about the human organism we did not knew before.

## Data Availability Statement

The original contributions presented in the study are included in the article/supplementary material, further inquiries can be directed to the corresponding author/s.

## Author Contributions

All authors conceived the research project and wrote the paper. All authors contributed to the article and approved the submitted version.

## Conflict of Interest

The authors declare that the research was conducted in the absence of any commercial or financial relationships that could be construed as a potential conflict of interest.
